# Competition of two highly specialized and efficient acetoclastic electroactive bacteria for acetate in biofilm anode of microbial electrolysis cell

**DOI:** 10.1038/s41522-021-00218-3

**Published:** 2021-05-31

**Authors:** Veerraghavulu Sapireddy, Krishna P. Katuri, Ali Muhammad, Pascal E. Saikaly

**Affiliations:** Biological and Environmental Science and Engineering Division, Water Desalination and Reuse Center, King Abdullah University of Science and Technology, Thuwal, 23955-6900 Saudi Arabia

**Keywords:** Water microbiology, Applied microbiology

## Abstract

Maintaining functional stability of microbial electrolysis cell (MEC) treating wastewater depends on maintaining functional redundancy of efficient electroactive bacteria (EAB) on the anode biofilm. Therefore, investigating whether efficient EAB competing for the same resources (electron donor and acceptor) co-exist at the anode biofilm is key for the successful application of MEC for wastewater treatment. Here, we compare the electrochemical and kinetic properties of two efficient acetoclastic EAB, *Geobacter sulfurreducens* (GS) and *Desulfuromonas acetexigens* (DA), grown as monoculture in MECs fed with acetate. Additionally, we monitor the evolution of DA and GS in co-culture MECs fed with acetate or domestic wastewater using fluorescent in situ hybridization. The apparent Monod kinetic parameters reveal that DA possesses higher *j*_max_ (10.7 ± 0.4 A/m^2^) and lower *K*_S, app_ (2 ± 0.15 mM) compared to GS biofilms (*j*_max_: 9.6 ± 0.2 A/m^2^ and *K*_S, app_: 2.9 ± 0.2 mM). Further, more donor electrons are diverted to the anode for respiration in DA compared to GS. In acetate-fed co-culture MECs, DA (98% abundance) outcompete GS for anode-dependent growth. In contrast, both EAB co-exist (DA: 55 ± 2%; GS: 24 ± 1.1%) in wastewater-fed co-culture MECs despite the advantage of DA over GS based on kinetic parameters alone. The co-existence of efficient acetoclastic EAB with high current density in MECs fed with wastewater is significant in the context of functional redundancy to maintain stable performance. Our findings also provide insight to future studies on bioaugmentation of wastewater-fed MECs with efficient EAB to enhance performance.

## Introduction

The current trend in biological wastewater treatment is moving toward recovery of resources (e.g., reclaimed water for reuse, energy, nutrients, materials, etc.)^1,2^. In this regard, anaerobic biotechnologies based on microbial electrochemical technologies (METs) have the potential for recovering energy and reclaimed water (for non-portable use) from domestic wastewater^3,4^. Recently, METs have been demonstrated to be efficient in treating domestic wastewater at a cubic-meter scale^5^. In METs, electroactive bacteria (EAB) are considered the key functional microbiome responsible for transforming organic (e.g., acetate) or inorganic (e.g., ammonium) pollutants in wastewater into energy through the electrogenesis process at the anode^6–8^. This is possible because EAB have extracellular electron transfer (EET) capability that allow them to couple the oxidation of substrates (electron donor) in their cytoplasm with the reduction of insoluble extracellular electron acceptors (e.g., electrode) for respiration^9^. Since EAB represent the core of METs, maintaining functional stability in terms of coulombic efficiency (CE), current density, and pollutant removal depends on maintaining diverse and efficient EAB on the anode biofilm^10^.

In engineered biological wastewater treatment systems, functional stability is often correlated with functional redundancy. Functional redundancy enables the maintenance of stable reactor performance as conditions change due to the presence of multiple species that can carry out the same biochemical function (e.g., phosphorous removal, ammonium oxidation, nitrite oxidation, etc.) such that the loss or change in the relative abundance of one species will be compensated by another species in the community^11–13^. In METs, identifying functionally redundant EAB is complicated as currently there is no marker gene to detect EAB like the genetic markers for well-studied microbes such as methanogens, ammonia-oxidizing bacteria, nitrite-oxidizing bacteria, and phosphorous accumulating organisms^14,15^. Also, there is no database available with known genes/proteins and pathways involved in EET, and therefore using “omics analyses” alone cannot determine if an organism is electroactive or not. Further, a literature survey of more than 100 EET-capable species indicated that there are many ecological niches for microorganisms able to perform EET^14^. Despite having EET capability, these known EET-capable species differ in their electron transfer capacity (direct vs. mediated electron transfer), habitat (oxygen, salinity, temperature, and pH), growth characteristics including the ability to form biofilm on the anode, and metabolic versatility (electron donors, electron acceptors, and carbon source). Therefore, relying solely on EET capability is not a criterion to ensure functional redundancy and hence functional stability of METs for domestic wastewater treatment. To maintain functional stability, EAB should be functionally redundant in terms of maintaining high current density and efficiency in converting substrates to current (i.e., high CE) at all times. This can be achieved by selecting functionally redundant EAB having a similar ecological niche (e.g., they are efficient in their EET, can generate high current density through direct electron transfer, can form a biofilm, and have similar metabolic characteristics).

Some EAB are known for being more flexible and versatile (i.e., occupy a wider ecological niche), while others are highly specialized and occupy a small ecological niche^14^. From the perspective of wastewater treatment with resource recovery, EAB that are highly specialized seem to be more efficient in terms of CE and current density. For instance, *Geobacter sulfurreducens*, a model EAB, is highly specialized and is currently considered the most important current-producing bacterium, and it is the most commonly identified anodic EAB derived from municipal wastewater in microbial fuels cells (MFCs) or microbial electrolysis cells (MECs), using acetate containing growth medium^6,7,15,16^. *G. sulfurreducens* interact with the anode by direct electron transfer using outer membrane cytochromes^17^ and nanowires^18^ to externalize the electrons from cell to the anode. They are metabolically less versatile and preferentially metabolize acetate (electron donor) with anode as electron acceptor and could form biofilm. Acetate is an important volatile fatty acid in domestic wastewater generated from the fermentation of organics, and serves as a primary substrate for EAB in METs^7^. Further, low acetate concentrations are shown to impose a selective pressure to enrich for *Geobacter* sp.^6,19^. The above features provide a selective advantage for the growth of *G. sulfurreducens* on the anode over more flexible and metabolically versatile EAB such as *Shewanella oneidensis* (another model EAB), and it also gives them a competitive advantage over non-EAB such as acetoclastic methanogens.

Despite the above advantages, *G. sulfurreducens* is not always present or abundant in METs fed with acetate^20^ or wastewater^10^. For instance, using acetate as electron donor and poised anode as electron acceptor, highly efficient EAB were selected from seven environmental samples to produce high current densities in MECs. *Geobacter* sp. was only dominant in two of the anodic biofilms^20^. In another study, electricity-generating biofilm was functionally stable for over 1 year in single-chamber, air-cathode MFCs fed with domestic wastewater, despite temporal fluctuations in a microbial community, with early stages dominated by *Geobacter* sp., but in later stages (mature biofilm), members closely related to *Desulfuromonas acetexigens* were predominant. Similarly, a shift in dominance from *Geobacter* sp. towards members closely related to *D. acetexigens* in the anodic biofilms was observed in single-chambered MECs (~14 days of growth using anaerobic sludge as inoculum) fed with acetate^21^. *D. acetexigens* was also detected along with *G. sulfurreducens* in the anodic biofilms enriched from mixed-culture inoculums, such as anaerobic sludge^21^, domestic sewage^10,22^, raw paper mill effluents^23^, and lagoon sediment^24^. Recently, a pure culture of *D. acetexigens* strain 2873 was shown to be capable of EET to the anode of MEC fed with acetate, producing high peak current densities of ~10 A/m^2^ ^21,25^. Taken together, these results suggest that other efficient acetoclastic EAB such as *D. acetexigens* might become dominant at the anode when conditions are favorable, hence supporting functional stability through functional redundancy. However, it is still not clear what triggers the selection of *G. sulfurreducens* or *D. acetexigens* to become dominant at the anode of METs fed with acetate or domestic wastewater.

From the perspective of ecological niche, *D. acetexigens* has a similar ecological niche as *G. sulfurreducens* in terms of electron transfer characteristics (i.e., direct vs. mediated) and metabolic characteristics (electron donor and acceptor). For example, they both preferentially metabolize acetate (electron donor) with anode as electron acceptor and could form biofilm. They also produce high current density through direct electron transfer. Nevertheless, they might differ in the genes/proteins and pathways involved in EET and in their growth and substrate-utilization kinetics. From the limited studies available in mixed-culture anodic biofilms^21^, it seems that *D. acetexigens* has some selective advantage over *G. sulfurreducens*. However, these studies are not conclusive because of the complexity of undefined mixed cultures. This complexity can be reduced using defined co-culture experiments in METs^26,27^. Previous co-culture experiments were conducted using mixed-substrates (acetate and lactate) as a source of carbon and energy and a co-culture made of a highly specialized and efficient EAB (*G. sulfurreducens*) and metabolically versatile and poorly efficient EAB (e.g., *S. oneidensis*, *Clostridium acetobutylicum*, or *Enterococcus faecium*)^26,27^. To the best of our knowledge, there are no co-culture studies that investigated the competition of highly specialized and efficient EAB for the same resources (electron donor and acceptor) in METs.

In domestic wastewater, daily fluctuations in wastewater composition, substrate complexity, and fermentation kinetics in the anode chamber is considered to be the rate-limiting step for electrogenesis^28^. Moreover, the process of fermentation of organics to acetate may vary temporally and spatially across the anode, which can lead to notable differences in acetate flux in the anodic chamber. Also, acetate can serve as a primary substrate for other competing processes (i.e., methanogenesis and sulfate reduction) in wastewater-fed reactors^28^, which might affect the enrichment of acetoclastic EAB as well as their activity at the anode (i.e., current density and CE). Therefore, for the successful application of METs for domestic wastewater treatment, it is important to first investigate whether highly specialized and efficient acetoclastic EAB competing for the same resources (electron donor and acceptor) co-exist in the anode biofilm. This motivated us to address the following questions in this study: will competition for a common electron donor (i.e., acetate) and electron acceptor (i.e., anode) in the anode biofilm lead to competitive exclusion or co-existence of efficient acetoclastic EAB? Is it possible to establish and maintain functional redundancy of efficient acetoclastic EAB in the anodic biofilm of MET treating domestic wastewater? To address these questions, we compared the electrochemical and kinetic properties of two efficient and highly specialized acetoclastic EAB, *Desulfuromonas acetexigens* and *Geobacter sulfurreducens*, grown as monoculture under identical operational conditions in single-chamber MECs. Then, we investigated the evolution of *D. acetexigens* and *G. sulfurreducens* in the anodic biofilm of MECs fed with acetate or domestic wastewater using fluorescence in situ hybridization (FISH).

## Results

### MEC performance with a monoculture of *D. acetexigens* or ***G. sulfurreducens***

**Fig. 1:**
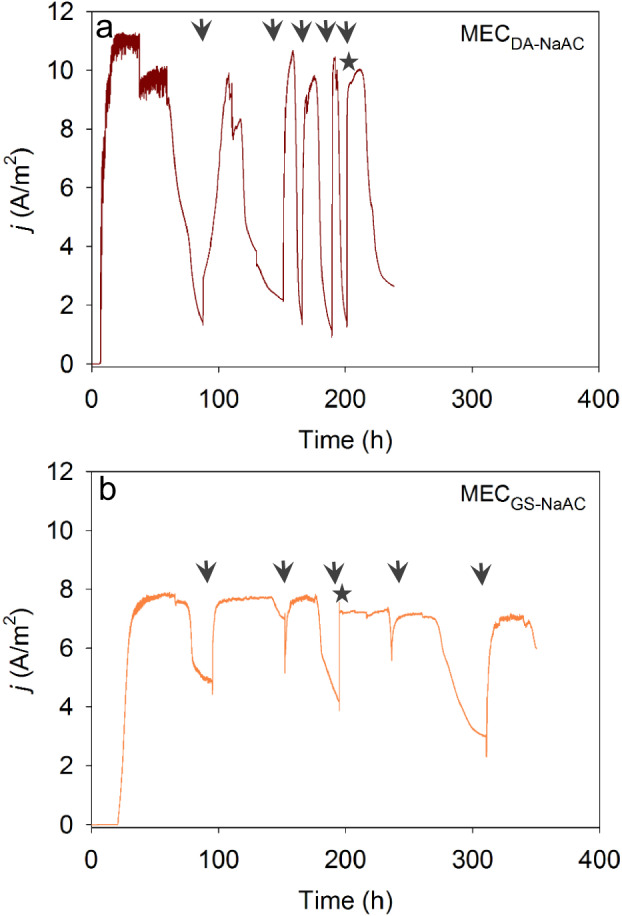
Amperometric response of biofilms grown on carbon cloth anode under a set anode potential of −0.1 V vs. Ag/AgCl and with acetate as the sole carbon and energy source. Amperometric response of **a** *D. acetexigens* (MEC_DA-NaAc_) and **b** *G. sulfurreducens* (MEC_GS-NaAc_) anode biofilms. Arrows indicate the change of feed. The first two batches were fed with 10 mM acetate containing growth medium, and the remainder of the batches were fed with 6 mM acetate. The asterisk indicates the time when the biofilms were subjected to cyclic voltammetry analysis. The current densities represent the average of triplicate MECs.

*D. acetexigens* (MEC_DA-NaAc_) and *G. sulfurreducens* (MEC_GS-NaAc_) biofilms were grown separately on graphite cloth under an anode set potential of −0.1 V vs. Ag/AgCl in single-chambered MECs (Supplementary Fig. 1). The cell density to startup the MECs was the same for both cultures, and acetate was used the sole carbon and energy source for reactor operation. The initial two batches were operated with 10 mM acetate (COD equivalent of 630 mg COD/L) to avoid any substrate limitation during initial anode colonization, and then the acetate concentration was reduced to 6 mM (COD equivalent of 380 mg COD/L) for the remainder of the batches to maintain a COD level typical of domestic wastewater. The anodic (i.e., chronoamperometry) response of the respective cultures (Fig. 1) revealed that the onset of a rapid increase in current started earlier in MEC_DA-NaAc_ (approximately at 10 h) than MEC_GS-NaAc_ (~at 20 h) following reactor inoculation (i.e., startup). In the 1st batch of operation, a peak current density of 11 ± 0.2 A/m^2^ was observed in MEC_DA-NaAc_, which is ~1.4 times higher compared to MEC_GS-NaAc_ (8 ± 0.4 A/m^2^). Chronoamperometry for the initial two batches using 10 mM acetate was sustained for a longer time (~70–100 h depending on the batch cycle) for both cultures compared to batch 3 and onwards (MEC_DA-NaAc_: 31 ± 5 h and MEC_GS-NaAc_: 52 ± 7 h) when the MECs were fed with 6 mM acetate. A slight decrease in peak current density was observed by reducing the acetate concentration from 10 mM (MEC_DA-NaAc_: 11 ± 0.1 A/m^2^ and MEC_GS-NaAc_: 8 ± 0.5 A/m^2^) to 6 mM (MEC_DA-NaAc_: 9.9 ± 0.3 A/m^2^ and MEC_GS-NaAc_: 7.2 A/m^2^). High CE was observed for both MEC_DA-NaAc_ (98 ± 2%) and MEC_GS-NaAc_ (>110 ± 10%) for the last three reproducible cycles of the current generation.

The difference in the magnitude of the current generation and batch duration between MEC_DA-NaAc_ and MEC_GS- NaAc_ was further investigated by looking closer at the profile of acetate consumption during batch 6 of operation (Supplementary Fig. 2). In both reactors, the acetate removal rate followed first-order kinetics with respect to acetate concentration with removal rate constant higher for MEC_DA-NaAc_ (0.076 ± 0.002 h^−1^) than MEC_GS-NaAc_ (0.065 ± 0.001 h^−^^1^). Rapid acetate consumption was noticed in MEC_DA-NaAc_ (82 ± 2% removal) than in MEC_GS-NaAc_ (68 ± 3% acetate removal) during the first 5 hours of batch operation. In MEC_DA-NaAc_, a sharp drop in peak current generation was noticed after 12 h of batch operation when the acetate concentration reached ~1 mM (87 ± 3% of acetate removal). Similar acetate removal was achieved in MEC_GS-NaAc_ after 25 h of batch operation, but peak current generation continued even after ~90% removal of acetate (after 30 h of operation), possibly due to H_2_, which was generated through HER at the cathode, being consumed by *G. sulfurreducens* as an electron donor for current generation^29^, which resulted in prolonged batch duration with >100% CE^7,30^. To test this, H_2_ availability was avoided in one of the batches by continuously purging N_2_ gas in the headspace (Supplementary Fig 3A). In the absence of N_2_ purging, the peak current density was maintained even after the acetate concentration reached <1 mM. Also, a significant reduction in batch duration (~35 h) was noticed with a CE of ~90% with N_2_ purging compared to a batch duration of >40 h and CE > 100% with no N_2_ purging. It should be noted that the cathodic CE (CCE) for H_2_ measured at the end of a batch cycle was <50% in MEC_GS-NaAc_ compared to a CCE of ~93 ± 2% in MEC_DA-NaAc_, which further supports the ability of *G. sulfurreducens* to recycle H_2_ for the current generation. In single-chamber MECs, values of CEs >100% are typically reported in the literature due to H_2_ recycling^31–33^. Also, an increase in CE by 5–12% due to H_2_ recycling by *G. sulfurreducens* has been previously reported in MECs fed with acetate^34^.

**Fig. 2:**
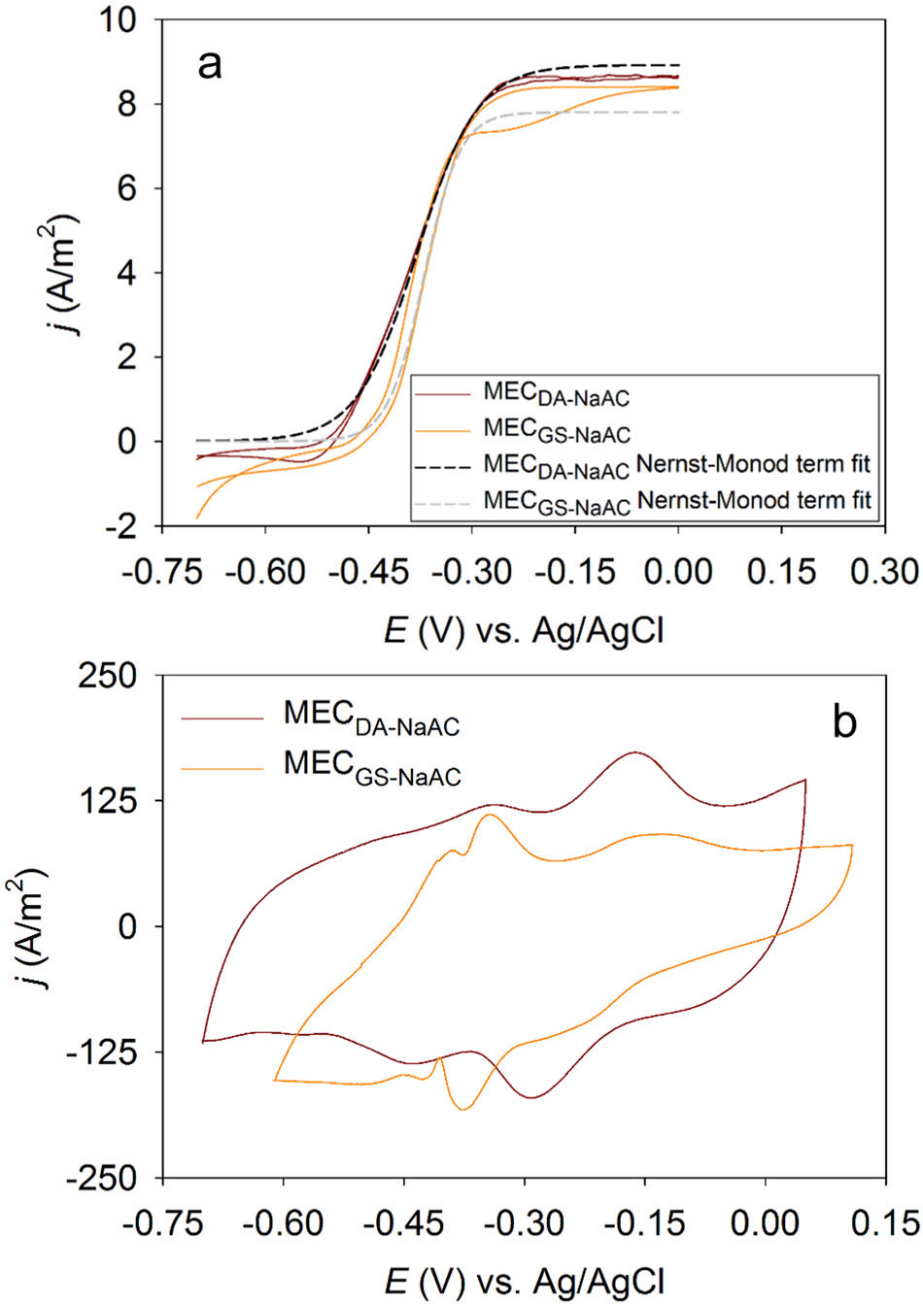
Slow scan (1 mV/s) cyclic voltammetry (CV) of *D.acetexigens* (ME_CDA-NaAc_) and *G. sulfurreducens* (ME_CGS-NaAc_) biofilms developed under a set anode potential of −0.1 V vs. Ag/AgCl for ~200 h. **a** CV recorded in the presence of 6 mM of acetate as the sole electron donor. The dashed curve lines in **a** represent the Nernst–Monod model fit for *n* = 1. **b** CV behavior of both biofilms under non-turnover conditions in the growth medium (lacking acetate).

The CV analysis of the ~200 h aged *D. acetexigens* and *G. sulfurreducens* biofilms developed in MEC_DA-NaAc_ and MEC_GS-NaAc_ showed a sigmoid shaped *j–E* curve that match the Nernst–Monod model (Fig. 2a), which is expected for electrocatalytic oxidation of acetate by the anode-bound EAB^35^. The *E*_KA_ (the anode potential giving one-half *j*_max_) for *D. acetexigens* biofilm anode was −0.37 V vs. Ag/AgCl, which was 10 mV more positive than *G. sulfurreducens* (*E*_KA_ = –0.38 V vs. Ag/AgCl) biofilm (Fig. 2a and Supplementary Fig. S4). Non-turnover CV (i.e., in the absence of electron donor) analysis of biofilms (Fig. 2b) in phosphate buffer (50 mM, pH 7.0) showed a clear difference in the voltammogram response for both cultures. Three distinct cell membrane-bound redox couples from the *D. acetexigens* biofilms are responsible for the current generation. The estimated mid-point redox potentials of these redox moieties were −0.58, −0.37, and –0.2 V vs. Ag/AgCl. However, *G. sulfurreducens* expressed four distinct redox moieties in the biofilms with redox potentials centered at −0.52, −0.4, −0.36, and −0.13 V vs. Ag/AgCl.

### MEC performance with a co-culture of *D. acetexigens* and *G. sulfurreducens*

Three sets of duplicate single-chambered MEC reactors were started simultaneously to evaluate the effect of co-culture (i.e., *D. acetexigens* and *G. sulfurreducens*) on MEC performance. The same cell density (~3E+08 cells/mL) was used for each culture to startup the reactors, which were fed with either a synthetic growth medium containing acetate or domestic wastewater amended with glucose to maintain a COD level of ~600 for the first two batches or 400 mg/L for batch 3 onwards. As with the monoculture MEC experiments, the initial two batches for the acetate-fed co-culture MECs were operated with 10 mM acetate (COD equivalent of 600 mg COD/L) to avoid any substrate limitation during initial anode colonization, and then the acetate concentration was reduced to 6 mM (COD equivalent of 380 mg COD/L) for the remainder of the batches.

**Fig. 3:**
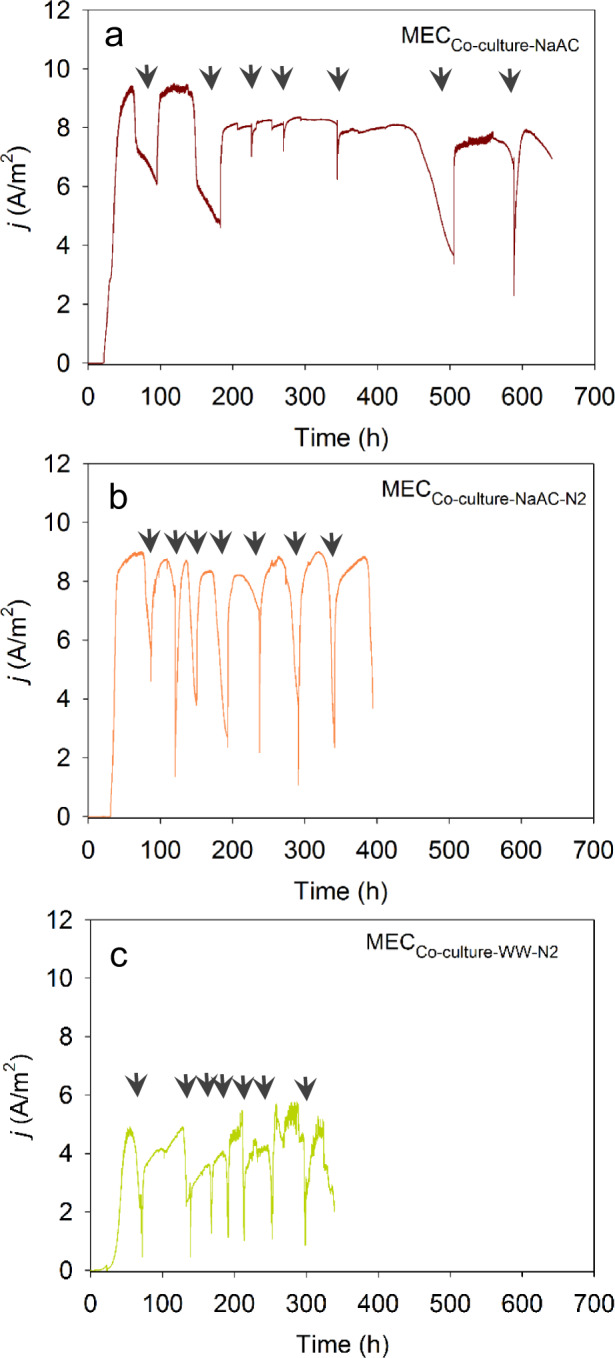
Amperometric responses for the co-culture MEC reactors. **a** Reactors fed with synthetic growth medium with sodium acetate as the sole carbon and energy source and operated without N_2_ purging in headspace (MEC_co-culture-NaAc_), **b** synthetic growth medium with sodium acetate as the sole carbon and energy source and operated with N_2_ purging in headspace (MEC_co-culture-NaAc-N2_), and **c** domestic wastewater and operated with N_2_ purging in headspace (MEC_co-culture-WW-N2_). The current densities represent the average of duplicate MEC reactors. Arrows indicates the change of feed. The first two batches of MEC_co-culture-NaAc_ and MEC_co-culture-NaAc-N2_ reactors were operated with 10 mM acetate containing growth medium (COD equivalent of 600 mg/L), and the remainder of the batches were operated with 6 mM acetate (COD equivalent of 400 mg/L). The first two batches of MEC_co-culture-WW-N2_ reactors were operated with glucose-supplemented domestic wastewater (COD equivalent of 600 mg/L), and the remainder of the batches were operated with glucose-supplemented domestic wastewater at a COD equivalent of 400 mg/L.

The profile of current generation in the reactors fed with synthetic medium did not vary much with (MEC_co-culture-NaAc-N2_) or without N_2_ (MEC_co-culture-NaAc_) purging in the headspace (Fig. 3a, b) with the onset and magnitudes of the current generation were more-or-less the same between the two sets of reactors. However, the time required for completion of a batch cycle was comparatively longer in MEC_co-culture-NaAc_ (>50 h; CE > 100%) than MEC_co-culture-NaAc-N2_ (~35 h, CE 90%) due to H_2_ recycling by *G. sullfurreducens*. This was confirmed experimentally where the time to see a drop in peak current production followed by a batch-cycle initiation in MEC_co-culture-NaAc_ was longer (>40 h) in the absence of N_2_ compared to the presence of N_2_ (30 h) in the reactor headspace even after the acetate concentration reached to <1 mM (Supplementary Fig. 3B).

When domestic wastewater was used as the main source of carbon and energy, the current generation in MEC_co-culture-WW-N2_ reactors was stabilized after four batches of reactor operation (Fig. 3c) with a CE of 39 ± 5% and COD removal of 88 ± 4%. However, the peak current generation (5 ± 0.9 A/m^2^) in MEC_co-culture-WW-N2_ (Fig. 3c) fed with domestic wastewater (containing fermentable substrates) was half the magnitude of peak current generated in MEC_co-culture-NaAc-N2_ (Fig. 3b) fed with acetate (a simple and non-fermentable substrate).

### Evolution of *D. acetexigens* and *G. sulfurreducens* in anode biofilm of co-culture MECs

**Fig. 4:**
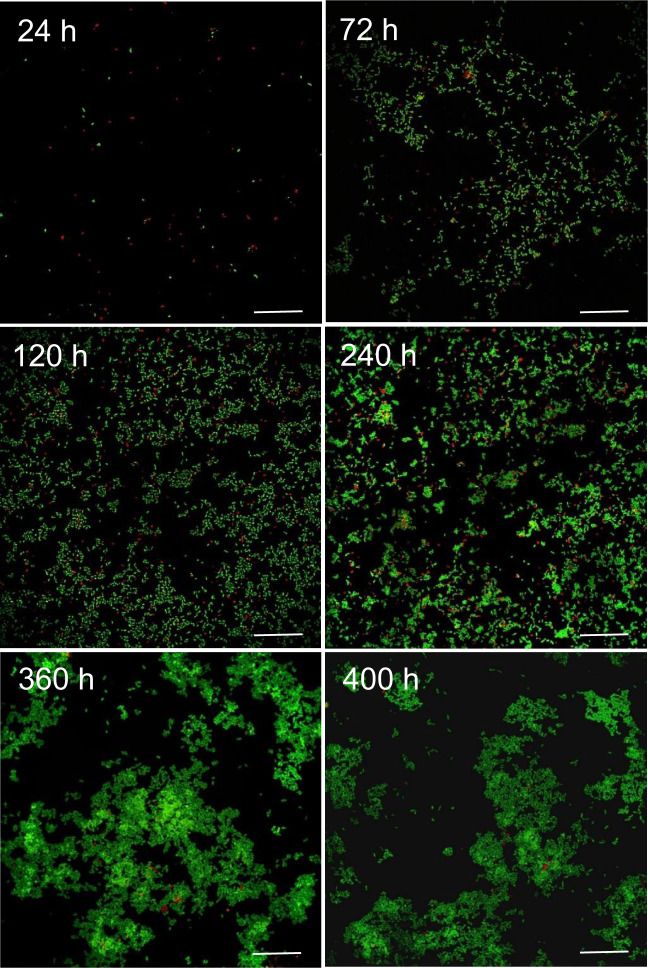
Representative FISH-CLSM images of *D. acetexigens* (green) and *G. sulfurreducens* (red). Cells extracted from the anode biofilm of a co-culture MEC reactor fed with synthetic medium containing acetate (MEC_co-culture-NaAc-N2_). Samples for FISH analysis were collected from the anode biofilm at different time periods of reactor operation. Scale bar for all images is 25 µm.

The evolution in the abundance of *D. acetexigens* and *G. sulfurreducens* over time in the anodic biofilms of co-culture MEC experiments using synthetic media (MEC_co-culture-NaAc-N2_) or domestic wastewater (MEC_co-culture-WW-N2_) was monitored by FISH-CLSM. To avoid the effect of H_2_ recycling from the cathode on the growth of *G. sulfurreduces* on the anode, FISH was mainly conducted for co-culture MEC reactors purged continuously with N_2_ in the headspace. The FISH results for the MEC reactors fed with acetate (MEC_co-culture-NaAc-N2_) showed that the number of *G. sulfurreduces* cells (8.5E + 04 cells/cm^2^) on the anode after 24 h of potential induced growth was 3 folds higher than *D. acetexigens* (2.8E + 04 cells/cm^2^) (Fig. 4 and Supplementary Table 1). However, the situation was reversed between hours 72 and 480 where the number of *D. acetexigens* cells was significantly higher (increasing from 9 folds at 72 h to 51 folds at 480 h; *P* = 0.007) than *G. sulfurreducens* (Supplementary Table 1). The high relative abundance (~98%) of *D. acetexigens* suggests that current generation was mainly attributed to the electrogenic activity of *D. acetexigens* in the anodic biofilm.

**Fig. 5:**
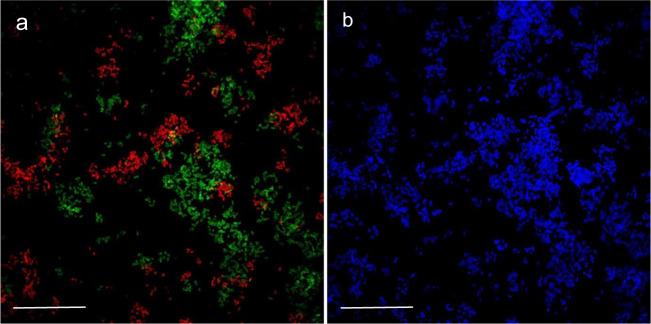
Representative FISH image of *D. acetexigens* (green) and *G. sulfurreducens* (red). **a** Cells extracted from the anode biofilm of a co-culture MEC reactor fed with glucose-supplemented domestic wastewater (MEC_co-culture-WW-N2_). **b** Total bacterial cells stained with DAPI (blue). The sample was collected after 360 h of reactor operation. Scale bar on images **a** and **b** is 25 µm.

As observed in the MEC reactors fed with acetate, the relative abundance of *D. acetexigens* was significantly higher (44 ± 3.6% at 120 h and 55 ± 2% at 360 h) than *G. sulfurreduces* (21.7 ± 0.7% at 120 h and 24 ± 1.1% at 360 h) in the MEC reactors fed with domestic wastewater (MEC_co-culture-WW-N2_) (Supplementary Table 2). While the influent wastewater feed resulted in other microorganisms growing in the mixed-culture anodic biofilm (Fig. 5), the combined relative abundance of *D. acetexigens* and *G. sulfurreducens* increased from 66 ± 2.8% (120 h) to 79 ± 3.1% (360 h) (Supplementary Table 2). It should be noted that both *D. acetexigens* and *G. sulfurreducens* were not detected by FISH in the influent wastewater feed. The enrichment of other bacteria (20% of non-FISH probe targeted cells) on the anode suggest that a portion of the COD was diverted for supporting their growth, which explains the low CE (39%) using domestic wastewater compared to acetate containing growth medium^36^. The acetate concentration detected during a batch cycle of operation of MEC_co-culture-WW-N2_ reactor was ≤0.5 mM (Supplementary Fig. 5). The pH of the electrolyte in the MECs ranged between 7.0 and 7.5 throughout the operation period. This pH is optimal for the growth of both *D. acetexigens*^21^ and *G. sulfurreducens*^37^.

### Estimation of apparent kinetic parameters for *D. acetexigens* and G. sulfurreducens in a biofilm anode

**Fig. 6:**
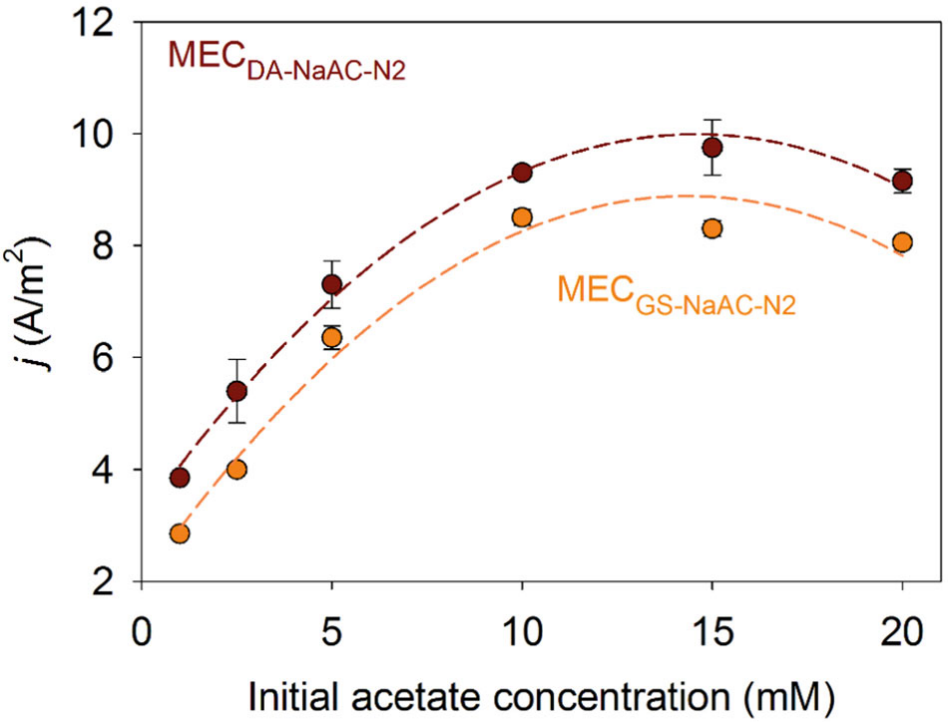
Plot of peak current densities vs. acetate concentrations for *D. acetexigens* (MEC_DA-NaAc_) and *G. sulfurreducens* (MEC_GS-NaAc-N2_). The dashed curve lines represent the Monod equation (Eq. 1) plots using the apparent kinetic parameters (*j*_max_ and *K*_S, app_) calculated for *D. acetexigens* and *G. sulfurreducens*. Results from duplicate MECs are represented as mean ± SD.

The effect of limiting-substrate concentration (1–20 mM of acetate) on current density under a set anode potential of −0.1 V vs. Ag/AgCl was evaluated for 200 h-aged biofilms of *D. acetexigens* and *G. sulfurreducens* in duplicate MEC_DA-NaAc-N2_ and MEC_GS-NaAc-N2_ reactors. The peak current densities generated at different acetate concentrations were used to estimate the apparent kinetic parameters (*j*_max_ and *K*_S, app_) for *D. acetexigens* and *G. sulfurreducens*. Results suggested that both cultures exhibited a Monod-like saturation curve (Fig. 6) as reported earlier for other electrochemically active bacteria^35,38^, with higher *j*_max_ (10.7 ± 0.4 A/m^2^) and lower *K*_S, app_ (2 ± 0.15 mM) estimated for *D. acetexigens* than *G. sulfurreducens* biofilms (*j*_max_: 9.6 ± 0.2 A/m^2^ and *K*_S, app_: 2.9 ± 0.2 mM). The lower *K*_S, app_ value for *D. acetexigens* compared to *G. sulfurreducens* suggest that *D. acetexigens* has a higher affinity for acetate than *G. sulfurreducens*.

**Fig. 7:**
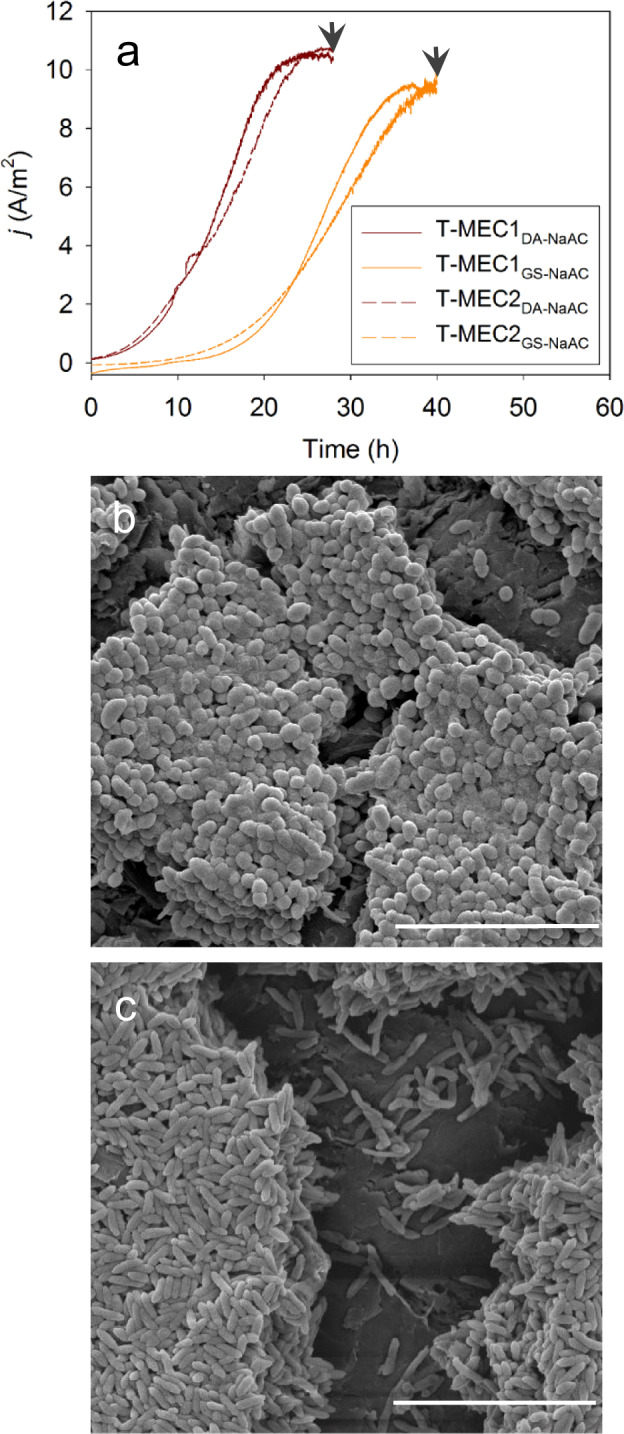
Amperomteric response and biofilm morphology during the early-stage of biofilm formation. **a** Amperometric response of *D. acetexigens* (T-MEC_DA-NaAc_) and *G. sulfurreducens* (T-MEC_DA-NaAc_) biofilms during the initial phase of biofilm development. Duplicate reactors were operated for each monoculture. Arrows in **a** indicate the time when the biofilms were subjected for scanning electron microscope (SEM) imaging and biokinetic parameters assessment. SEM images of **b** *D. acetexigens*, and **c** *G. sulfurreducens*. Scale bar on images **b** and **c** is 10 µm.

To estimate *µ*_max_ (d^−1^), Y_net_ (g VSS per g COD) and *f*_s_, a new set of duplicate two-chambered MEC reactors (T-MEC_GS-NaAc_ and T-MEC_DA-NaAc_) were initiated using previously acclimatized *D. acetexigens* (MEC_DA-NaAc_) and *G. sulfurreducens* (MEC_GS-NaAc_) anode biofilms as the inoculum (1% v/v, ~2E+08 cells/ml). The reactors were operated under non-substrate (10 mM acetate) limiting conditions at an anode set potential of −0.1 V vs. Ag/AgCl. The initial concentration of acetate (10 mM) was much higher than the calculated *K*_S, app_ for both cultures. The SEM images of anode colonized biofilms of representative T-MEC_DA-NaAc_ and T-MEC_GS-NaAc_ reactors clearly represent the morphological features of *D. acetexigens* (Fig. 7b) and *G. sulfurreducens* (Fig. 7c). The T-MEC_GS-NaAc_ reactor took longer time (40 h) to produce a peak current of 9.4 ± 0.1 A/m^2^ after inoculation compared to T-MEC_DA-NaAc_ reactor (10.6 ± 0.1 A/m^2^ after 25 h) (Fig. 7a). Higher biomass density and acetate consumption were measured for T-MEC_GS-NaAc_ (0.94 g protein/cm^2^ and 7.2 mM acetate) than T-MEC_DA-NaAc_ (0.66 g protein/cm^2^ and 6.4 mM acetate) to achieve peak current generation after inoculation. The *µ*_max_ (Supplementary Fig. 6), Y_net_, and *f*_s_ were 9 ± 1.7 d^−^^1^ (doubling time of 1.9 ± 0.3 h), 0.065 ± 0.002 g VSS per g COD, and 0.091 ± 0.003 *e*^-^ eq of biomass per *e*^-^ eq of donor consumed for T-MEC_GS-NaAc_, and 27.9 ± 4.8 d^−1^ (doubling time of 0.6 ± 0.1 h), 0.051 ± 0.001 g VSS per g COD and 0.072 ± 0.002 *e*^-^ eq of biomass per *e*^−^ eq of donor consumed for T-MEC_DA-NaAc_.

## Discussion

In this study, we compared the electrochemical and kinetic properties of two efficient acetoclastic EAB, *D. acetexigens* and *G. sulfurreducens*, grown as monoculture under identical operational conditions in single-chamber MECs. Additionally, we monitored the evolution of *D. acetexigens* and *G. sulfurreducens* in co-culture MECs fed with synthetic media (containing acetate) or domestic wastewater supplemented with glucose.

The chronoamperometric response observed in the monoculture experiments for *D. acetexigens* in MEC_DA-NaAc_ was different from *G. sulfurreducens* (MEC_GS-NaAc_) although both MECs were operated under the same conditions and started with the same inoculum cell density (Fig. 1). Noticeable differences were observed in the lag-period for current generation during reactor start-up. Also, the profile of current density, the magnitude of the current generation, and the time required to complete the individual batch of operation was different between the two EAB (Fig. 1). This difference in behavior between the two EAB could be due to differences in their (i) anode interaction mechanism (Fig. 2b), and (ii) apparent kinetic parameters in a biofilm anode (Fig. 6 and Supplementary Fig. 6), which are elaborated below.

The CV for turnover electron transfer (in the presence of acetate) of MEC_DA-NaAc_ biofilms retained the characteristic *j–E* response (Fig. 2a) similar to *G. sulfurreducens* biofilms developed in this study (i.e., MEC_GS-NaAc_) and in previous studies with mono-cultures of EAB^35,39,40^ or mixed cultures^20,33,41,42^. The CV analysis in the presence of acetate (Fig. 2a) indicates that the dominant redox-active protein(s) with a mid-point potential of approximately −0.37 to 0.38 V vs. Ag/AgCl at pH ~7.0 are responsible for efficient microbial-electrocatalytic electron transfer (i.e., *j*_max_) in the tested EAB. However, the expressed redox moieties from the non-turnover CV analysis of *D. acetexigens* biofilms appeared to differ from *G. sulfurreducens* biofilms (Fig. 2b). Differences in the number of expressed membrane-bound redox couples with distinct mid-point potentials between both current generating biofilms (Fig. 2b) indicate that the EET mechanism of *D. acetexigens* differs from *G. sulfurreducens*. Compared to *D. acetexigens*, the EET mechanism of *G. sulfurreducens* has been extensively studied^15^. Like *G. sulfurreducens* catalytic biofilms, *D. acetexigens* has the tendency to express biofilm-confined redox proteins (Fig. 2b) to generate current following the oxidation of the substrate (Fig. 2a). However, the non-turnover CV response of *D. acetexigens* biofilm-confined redox proteins (Fig. 2b) and their superior electrocatalytic activity (Figs. 1a and 6) implies that *D. acetexigens* may possess a unique EET pathway to interact with the anode. Future studies should employ various omic approaches to better understand the genes/proteins responsible for EET in *D. acetexigens*^8,43,44^.

In single-chamber MEC_S_, H_2_ recycling affects energy consumption and energy recovery (i.e., H_2_) from the system^3^. *G. sulfurreducens* is known to utilize H_2_ as an electron donor for electricity generation^29^. As expected, H_2_ recycling in the MEC_GS-NaAc_ reactors prolonged the time needed for completing the batch cycle. In contrast, the batch cycle was shorter in MEC_DS-NaAc_ because of the lack of H_2_ recycling. The inability of *D. acetexigens* to use H_2_ as an electron donor for elemental sulfur (as an electron acceptor) reduction was also reported earlier^45^. This further supports their inability to use H_2_ as an electron donor. This unique property (i.e., lack of H_2_-recycling ability) of *D. acetexigens* will allow its application in single-chamber MECs with maximum recovery of energy in form of H_2_.

The good fittings of the experimental *j–E* curves measured in electron turnover conditions to the Nernst–Monod model (Fig. 2a) suggest that bacterial kinetics controlled the *j–E* response^40^. Further, the good fittings indicate that *D. acetexigens* and *G. sulfurreducens* biofilm anodes having high current density were highly conductive^46^. Plotting the two Nernst–Monod curves (Supplementary Fig. 4) using the Nernst–Monod kinetics (*E*_KA_ and *j*_max_) of both acetoclastic EAB suggests that the anode potential (electron acceptor) does not provide a selective advantage for one electroactive bacterium over the other. Moreover, the anode-imposed potential of −0.1 V vs. Ag/AgCl (Supplementary Fig. 4) was not limiting the respiration of both EAB. These results suggest that other factors, such as differences in EET mechanism, might have been responsible for the higher *j*_max_ (Figs. 1 and 6) of *D. acetexigens* over the benchmark electroactive bacterium *G. sulfurreduces*.

In co-cultured reactors fed with acetate, the relative abundance of *G. sulfurreducens* was higher compared to *D. acetexigens* in the early-stage biofilms (after 24 of startup; *P* < 0.01) (Supplementary Table 1). However, this dominance immediately shifted towards *D. acetexigens* after 72 h of reactor operation and was maintained for the remainder of the experiment. While *G. sulfurreducens* was consistently present at low abundance (~2%) with *D. acetexigens*, we can assume that current production from acetate-fed MECs was mainly attributed to *D. acetexigens* activity due to its high abundance (~98%) in the anodic biofilm (Supplementary Table 1). The selective advantage of *D. acetexigens* over *G. sulfurreducens* could be due to differences in their EET mechanism and kinetic parameters (*j*_max_, *K*_S, app_, *µ*_max_, and *Y*_net_). Assuming no other parameters other than the electron donor is rate-limiting for the entire process of metabolism and EET, the simulated current density of *D. acetexigens* and *G. sulfurreducens* as a function of acetate concentration using the Monod equation (Eq. 1) and calculated kinetic parameters (*j*_max_, *K*_S, app_) demonstrated that *D. acetexigens* can yield higher current density compared to *G. sulfurreducens* at all soluble limiting-substrate concentrations (Supplementary Fig. 7). Further, lower *Y*_net_ and *f*_s_ values for *D. acetexigens* compared to *G. sulfurreducens* suggest that comparatively more donor electrons were diverted to the anode for respiration, whereas more donor electrons were diverted to cell synthesis in *G. sulfurreducens*. The above kinetic advantages provided a selective advantage for *D. acetexigens* over *G. sulfurreducens* resulting in a higher relative abundance of *D. acetexigens* in the biofilm of co-cultured MEC reactors fed with acetate.

In the case of co-cultured MEC reactors fed with domestic wastewater supplemented with glucose (MEC_co-culture-WW-N2_), the acetate concentration stabilized at 0.1–0.5 mM during the batch cycle of operation (Supplementary Fig. 5) suggesting that the rate of acetate generation by fermenters balanced the rate of its consumption by acetate-oxidizing bacteria, mainly *D. acetexigens* and *G. sulfurreducens*. This range of acetate concentration was significantly lower than the *K*_S, app_ for acetate (2–2.9 mM) for both organisms. However, if we assume that the biomass specific loss rate is the same (0.1 d^−1^) for both EAB, then *S*_min_ = *K*_S, app_ × (biomass specific loss rate/*µ*_max_ – biomass specific loss rate), which is defined as the minimum substrate concentration that can support steady-state biomass^13^, for *D. acetexigens* is 0.01 mM and 0.03 mM for *G. sulfurreducens*. This suggests that these low acetate concentrations could sustain the growth of both EAB. Contrary to acetate-fed co-culture MECs (MEC_co-culture-NaAc-N2_), where competitive exclusion prevailed with *D. acetexigens* (98% abundance) outcompeting *G. sulfurreducens* in a very short period (5 days) after reactor startup (Supplementary Table 1), both EAB co-existed at high relative abundance (*D. acetexigens* 55 ± 2%; *G. sulfurreducens* 24 ± 1.1%) in MEC_co-culture-WW-N2_ reactors (Supplementary Table 2) despite the selective advantage of *D. acetexigens* over *G. sulfurreducens* based on kinetic parameters alone. Such co-existence without competitive exclusion requires niche differentiation. Presumably, *G. sulfurreducens* co-existed due to the fermentable nature of the substrate (domestic wastewater and glucose), which requires syntrophic cooperation between fermenters and EAB for its degradation^6,16^.

The difference in the combined relative abundance of the two EAB at the anode when using acetate (100%) vs. wastewater (80–85%) was due to the nature of the substrate (fermentable vs. non-fermentable substrate). In wastewater-fed MECs, fermentable substrates are first fermented to acetate by fermenters (Supplementary Fig. 5) and the generated acetate is then utilized for current generation by the bio-augmented acetoclastic EAB. Also, the rate of fermentation, the spatial variability of acetate production on the anode by fermenters, and the presence of other heterotrophs competing for substrate and space in mixed-culture biofilms may have led to this difference in the relative abundance of bio-augmented EAB between the acetate-fed MECs and wastewater-fed MECs. Nevertheless, both EAB maintained high relative abundance (80–85%) on the anode of wastewater fed MECs after bioaugmentation due to several factors including their high affinity (low *K*_S, app_) to acetate (electron donor), low *S*_min_ for acetate, imposed growth at an anode potential of −0.1 V, and the fact that they both form biofilm and transfer electrons directly to the anode.

The co-existence of efficient acetoclastic EAB in MECs fed with real wastewater is significant in the context of functional redundancy for maintaining stable performance in response to environmental perturbations^11^. Although members of *Geobacter* sp. seem to be the dominant anodic community in lab-scale and pilot-scale MECs treating domestic wastewater^47–50^, the findings from this study suggest that *D. acetexigens* co-exists with *G. sulfurreducens* in MECs fed with domestic wastewater, and bioaugmentation of MECs with *D. acetexigens* and *G. sulfurreducens* might be a good strategy to enhance functional redundancy in the anodic biofilm and hence functional stability of MECs. Different EAB have different ecophysiological properties, and therefore, identifying factors (biotic and abiotic) that create ecological niches favoring the co-existence of superior acetoclastic EAB such as *D. acetexigens* and *G. sulfurreducens* in mixed-culture anodic biofilms warrants further investigation.

## Methods

### Pure culture growth

*G. sulfurreducens* (DSM 12127) and *D. acetexigens* (DSM 1397) were procured from the German Collection of Microorganisms and Cell Cultures (DSMZ, Germany) as lyophilized cultures and were activated separately in their respective culture medium as recommended by DSMZ. After activation, *G. sulfurreducens* and *D. acetexigens* were cultured separately at 30 °C in 50 mL airtight, rubber septa-sealed, anaerobic syringe bottles containing 45 mL of growth medium (details shown below) and subsequently sub-cultured four times (each batch was incubated for 3 days) in the same medium prior to their use in the MEC experiments. The growth medium contained (per liter): 0.1 g of KCl, 0.2 g of NH_4_Cl, 0.6 g of Na_2_HPO_4_, 0.49 g of CH_3_COONa (sole carbon and energy source), 10 mL of vitamin mix (DSM 141), and 10 mL of trace mineral mix (DSM 141). Followed by boiling, the medium was flushed with N_2_-CO_2_ (80:20) for 1 h to remove dissolved oxygen. Then the medium was adjusted to pH 7 by adding powdered NaHCO_3_, and then was transferred to serum vials for autoclaving (121 °C, 20 min, 15 psi). CaCl_2_ (final concentration of 0.1 g/L) and MgSO_4_ (final concentration of 0.4 g/L) solutions were autoclaved separately to avoid salt precipitation interference in the growth medium during heating, and were added to the sterilized growth medium prior to culture inoculation in MEC. Filter sterilized sodium fumarate solution (final concentration of 8 g/L, which was pre-dissolved in sterile degassed water) was added into the autoclaved medium as the terminal electron acceptor to initiate growth after inoculation. The final batch of inoculum sub-culturing was cultivated in 250 mL of serum vial containing 200 mL of growth medium to harvest sufficient biomass to conduct the MEC experiments. All transfers were carried out under an anaerobic environment.

### MEC construction and operation

Both cultures grown separately in 250 mL serum vials were centrifuged (7000 rpm for 8 min) and the resultant pellet from each culture was washed separately with saline phosphate buffer and centrifuged again. The washed cell pellets of each culture were suspended separately in sterile growth medium (free of fumarate). The cell concentration of individual cultures was normalized to ~3E + 08 live cells/mL, before inoculating single-chamber MECs, by adding a certain volume of the growth medium followed by measuring the cell density with flow cytometry.

The single-chamber MECs were constructed using screw-capped 300 mL borosilicate glass bottles and were operated with a working volume of 280 mL. Single-chamber MEC design was used in the current study because it is more practical for scale-up as it reduces capital and energy cost^43^. Also, single-chamber MECs minimize the pH imbalance that is typically observed between the anode (more acidic) and the cathode (more basic) in double-chamber MECs. The single-chamber MECs were modified with appropriate ports for placing electrodes, gas collection bag, and sampling liquid samples. The anode was carbon cloth (6 cm × 6 cm) with platinum gauze (5 cm × 5 cm) as cathode and Ag/AgCl as reference electrode (3.5 M KCl, BioAnalytical Systems, USA). The anode and cathode were positioned vertically, ~3 cm apart. The single-chamber MECs were operated under anodic potential induced growth of −0.1 V vs. Ag/AgCl using a multi-channel potentiostat (VMP3, Biologic, France). This potential was chosen for the following reasons: (i) in our previous study^21^, we showed that *D. acetexigens* generates high current density at −0.1 V vs. Ag/AgCl; and (ii) this anode potential falls between the optimum potential range (i.e., 0 to −0.2 V vs. Ag/AgCl) reported for *G. sulfurreducens* biofilm growth^51^.

Four sets of MEC experiments were conducted in parallel, with three sets operated using the above synthetic growth medium (free of fumarate) with sodium acetate (NaAc) as the sole carbon and energy source and one set was operated with unfiltered/unsterilized domestic wastewater as feed substrate. One set of triplicate MECs were started by inoculating a monoculture (2% culture) of *G. sulfurreducens* (MEC_GS-NaAc_) or *D. acetexigens* (MEC_DA-NaAc_) in separate MEC reactors. The second set of duplicate MECs were started by adding both cultures together (MEC_co-culture-NaAc_; 1% v/v of each culture). The MEC reactors were operated in a batch-cycle mode, and the feed substrate was changed when the current generation drops to half of its peak current value or when the measured residual acetate concentration becomes low (~1 mM). At the end of each batch of operation, the MEC reactors (monoculture or co-culture) were completely drained and then filled with fresh growth medium containing acetate, with no additional inoculum. The third set of duplicate reactors (MEC_co-culture-NaAc-N2_) was operated like the MEC_co-culture-NaAc_ but N_2_ gas was continuously purged in the reactor headspace. Concurrently, the fourth set of duplicate MEC co-culture reactors were operated using unfiltered and unsterilized domestic wastewater (MEC_co-culture-WW-N2_) as feed substrate (no acetate added) under N_2_ gas continuously purged in the reactor headspace. Both cultures (1% v/v of each culture) were added to the MEC_co-culture-WW-N2_ reactors. Domestic wastewater was collected from a local wastewater treatment plant (KAUST campus, Saudi Arabia), but due to the low organic strength (~150 mg/L of COD) of the wastewater, glucose (a fermentable substrate) was added to the wastewater to maintain a uniform COD concentration of 400 mg/L in the feed to match the COD level used in the acetate-fed MECs. Since competition for acetate is impacted by syntrophic interaction between EAB and fermenters when using fermentable substrates (e.g., real wastewater, glucose), we used glucose to mimic a fermentable substrate instead of acetate (non-fermentable substrate), otherwise adding acetate to adjust the COD will bias the results of competition using real wastewater. The first two batches of MEC_co-culture-WW-N2_ reactors were operated with 600 mg/L of COD to support microbial growth during the initial phase of anode colonization. All inoculations and media transfers were carried out in a sterile anaerobic glove box (Coy Laboratory, USA), and all incubations were performed at 30 °C in a controlled temperature room.

The biofilm from the co-culture MEC experiments (MEC_co-culture-NaAc-N2_ and MEC_co-culture-WW-N2_) was collected at different time intervals of reactor operation for cell count measurement using FISH.

### Apparent kinetic parameters estimation for *G. sulfurreducens* and *D. acetexigens* anodic biofilm

Two sets of duplicate MEC reactors (MEC_GS-NaAc-N2_ and MEC_DA-NaAc-N2_) were started by inoculating a monoculture (1% v/v; cell density of ~3.0E+08 cells/mL) of *G. sulfurreducens* or *D. acetexigens*. N_2_ gas was continuously purged in the headspace of the reactors through a 0.2 µm pore size sterilized filters to maintain uniformity in operation among the two sets of reactors. After observing reproducible current production followed by 200 h of biofilm growth under a set anode potential of −0.1 V vs. Ag/AgCl, the concentration of electron donor (acetate) was varied (1–20 mM) to estimate the apparent kinetic parameters (*j*_max_ and *K*_S, app_) according to Torres et al.^38,40,52^ using Monod equation (Eq. 1):

1 $$j = j_{\mathrm{max}}\frac{S}{{K_{{\mathrm{S}},{\mathrm{app}}} + S}}$$

Where, *j* is current density (A/m^2^), *j*_max_ is the maximum current density (A/m^2^) at the corresponding concentration of acetate (mM), *K*_S, app_ is the apparent half-saturation substrate concentration (mM) in a biofilm and *S* is the substrate (acetate) concentration (mM).

The *j*_max_ and *K*_S, app_ values for each monoculture were estimated using the relative least-square method^38^.

The maximum specific growth rate (*µ*_max_; d^−1^) and net biomass yield (*Y*_net_; g VSS per g COD) for *D. acetexigens* and *G. sulfurreducens* were estimated in separate two-chambered MEC experiments as described previously^46,53^. The *µ*_max_ was estimated by plotting the natural logarithm of *j* vs. time, according to Eq. 2, and then performing a linear regression for the initial growth phase:

2 $${\mathrm{In}}\left( {\frac{j}{{j_0}}} \right) = \mu _{\mathrm{max}}t$$

Where *j* and *j*_0_ represent the current densities produced at time *t* and *t* = 0, respectively.

The *Y*_net_ was estimated according to Eq. 3^53^:

3 $$Y_{\mathrm{{net}}}\left( {\frac{{{\mathrm{g}}\,{\mathrm{VSS}}}}{{{\mathrm{g}}\,{\mathrm{COD}}}}} \right) = \frac{{C_{{\mathrm{protein}}}\left( {\frac{{{\mathrm{g}}\,{\mathrm{protein}}}}{L}} \right)}}{{{\Delta}S_{{\mathrm{acetate}}}\left( {\frac{{{\mathrm{mole}}}}{L}} \right)}} \times \frac{{2\,{\mathrm{g}}\,{\mathrm{VSS}}}}{{{\mathrm{g}}\,{\mathrm{protein}}}} \times \frac{{1\,{\mathrm{mole}}}}{{64\,{\mathrm{g}}\,{\mathrm{COD}}}}$$

Where *C*_protein_ (g/L) is the concentration of protein measured at the end of the batch experiment, Δ*S*_acetate_ (mol/L) is the difference between influent and effluent acetate concentration, 64 is the COD equivalent of 1 mole of acetate. Total protein was measured using the DC-protein assay kit (BIO-RAD Laboratories, Inc., USA) following the manufacturer’s instructions after being re-suspended in deionized (DI) water, with a series of graded Bovine Serum Albumin (BSA, Sigma Aldrich, USA) solutions as standards^54^.

The fraction of donor electrons used for cell synthesis (*f*_s_) was calculated according to Eq. 4^53^:

4 $$f_{\mathrm{s}} = \frac{{Y_{{\mathrm{net}}}\left( {\frac{{{\mathrm{g}}\,{\mathrm{vss}}}}{{{\mathrm{g}}\,{\mathrm{COD}}}}} \right) \times \frac{{{\mathrm{mol}}_{{\mathrm{acetate}}}}}{{8\,e^ - {\mathrm{eq}}}} \times \frac{{64\,{\mathrm{g}}\,{\mathrm{COD}}}}{{{\mathrm{mol}}_{{\mathrm{acetate}}}}}}}{{113\left( {\frac{{{\mathrm{g}}\,{\mathrm{VSS}}}}{{{\mathrm{mole}}_{{\mathrm{cells}}}}}} \right) \times \frac{1}{{20}}\left( {\frac{{{\mathrm{mole}}_{{\mathrm{cells}}}}}{{e^ - {\mathrm{eq}}}}} \right)}} = 1.42Y_{{\mathrm{net}}}$$

where 8, 64, 113, and 20 are the number of electron equivalents in a mole of acetate, COD conversion factor for a mole of acetate, a molecular weight of bacterial cells according to the empirical formula of C_5_H_7_O_2_N, and the number of electron equivalents in a mole of biomass (with NH_4_^+^ as nitrogen source), respectively. The fraction of donor electrons used for energy generation (*f*_e_) via respiration was calculated as *f*_e_ = 1 − *f*_s_^46^.

### Electrochemical analysis

Cyclic voltammetry (CV) analysis at 1 mV/s was conducted for biofilm-covered anodes as working electrode when the electroactive bacterium (*G. sulfurreducens* or *D. acetexigens*) were oxidizing an electron donor (acetate), with platinum gauze cathode (2 × 2 cm) as counter electrode and Ag/AgCl as a reference electrode. Duplicate CV scans were performed for each electroactive bacterium and experimental CV was fit to Nernst–Monod equation with *n* = 1^46^. One MEC reactor from each of the triplicate MEC_GS-NaAc_ and MEC_DA-NaAc_ reactors was scarified for conducting the non-turnover CV analysis. Biofilm-covered anodes from both MECs (i.e., MEC_GS-NaAc_ and MEC_DA-NaAc_) were washed gently three times with saline sterile degassed phosphate buffer (pH 7.2) to remove any residual acetate in the biofilms. Then the reactors with acetate-free growth medium were incubated for 1 h under anaerobic conditions to further remove any residual acetate concentration in the biofilm matrix. Then the biofilms were polarized under a set potential of −0.1 V (vs. Ag/AgCl) for few minutes till the anodic current approached zero and then proceed for non-turnover CV analysis in the same cell at a scan rate of 1 mV/s. The CE was calculated as described earlier^55^.

### Acetate and H_2_ measurement

Acetate consumption rate was calculated using high-performance liquid chromatography (HPLC, Thermo-scientific) as previously described^3^. Hydrogen generated from the hydrogen evolution reaction (HER) at the cathode surface was measured as previously described^3^ using a gas chromatograph (GS, SRI Instruments).

### Flow cytometry

The bacterial cell count in the inoculum was measured by flow cytometry (BD Accuri C6 flow cytometer, BD Biosciences, Franklin Lakes, NJ) as previously described^36^. Samples (200 μL) were transferred to a sterile Eppendorf tube and incubated at 35 °C for 10 min prior to staining with SYBR Green I (2 μL of 100× stock solution in 200 μL sample), vortexed, and then incubated again at 35 °C for 10 min. Samples (200 μL) were then transferred to a 96-well plate for cell counting. Concurrently, another 200 µL of the samples were stained with propidium iodide (2 μL of 100× stock solution) in combination with SYBR Green I (2 μL of 100× stock solution) to find live and dead (membrane-compromised) cells according to the same protocol used for total bacterial cell count. A flow cytometer equipped with a 50 mW laser having a fixed emission wavelength of 488 nm was used to measure the cell count. Fluorescence intensity was collected at FL1 = 533 ± 30 nm, FL3 > 670 nm, sideward and forward scattered light intensities were obtained as well. Electronic gating was used to select SYBR green I as well as propidium iodide and SYBR Green I staining labeled signals for quantifying total bacterial as well as live and dead cells. All data were processed with the BD Accuri CFlow® software. Unless specified otherwise, the bacterial counts represent the live cell number.

### Scanning electron microscopy (SEM) and FISH

For SEM analysis, anode biofilms of MEC_DA-NaAc-N2_ and MEC_GS-NaAc-N2_ were fixed in 2% glutaraldehyde containing PBS (50 mM, pH 7.4) for 2 days at 4 °C. Then the biofilms were further processed for SEM imaging as reported earlier^4^.

For FISH analysis, biofilms (MEC reactors) from the co-culture experiments were collected at different time intervals for cell count measurements. Biofilms were randomly collected at regular intervals during reactor operation from the top, middle, and bottom of the anode using a sterilized scissor, and then each piece was transferred to separate 15 mL vials containing 5 mL of sterile extraction solution (phosphate buffer, pH 7.0, 10 mM). Following biofilm extraction through vigorous vortexing, the different anode pieces (2 × 2 cm) were washed (vigorously by vortexing for 10 s) separately in new vials having 5 mL of extraction buffer. Then this solution was pooled to the initial extracted biofilm vial, and centrifuged (8000 rpm for 6 min) to collect the extracted biofilm pellet. The resulting pellet was re-suspended in 5 mL of 8% (w/v) paraformaldehyde to fix the cells and incubated for 3 h at 4 °C^8^. Following incubation, the samples (biofilms and suspended cells) were gently washed twice with phosphate buffer saline (PBS 10 mM, pH 7.2) and then preserved in ethanol-PBS solution (1:1) in the freezer (−20 °C) until further processing with FISH

A previously designed FISH probe (5′-CTC ACG CAC TTC GGG ACC AA-3′) labeled with FITC was used to detect *G. sulfurreducens* cells^56^. The PROBE_DESIGN tool of the ARB software was used to design the FISH probe (5′-CGT CAG GCC CAG GCG ATA-3′) for detecting *D. acetexigens* cells and it was labeled with CY3. The hybridization efficiency of both FISH probes was tested and found to be optimal at 20% (w/v) formamide at 46 °C. The specificity of the FISH probes was tested in silico^57^ and experimentally using synthetic growth medium as well as domestic wastewater spiked with a known cell density of each culture. Also, non-spiked wastewater samples were used as a negative control. It should be noted that the *G. sulfurreducens* FISH probe did not target *D. acetexigens* cells and vice versa. Re-suspended samples (5 μL) were loaded into gelatin-coated wells and then the slides were dehydrated by sequential immersion for 3 min in 50%, 80%, and 100% ethanol and air-dried. For hybridization, 10 μL of hybridization buffer (720 μL of 5 M NaCl, 80 μl of 1 M Tris/HCL, 10% SDS, 20% (w/v) formamide, and filled to 4 mL with MilliQ water) containing 1 µL of each probe was added to the wells containing cells. The slides were incubated for 3 h at 46 °C. After hybridization, the cells were washed in a washing buffer (500 μL of 0.5 M EDTA, 1000 μL of 1 M Tris/HCL, 10% SDS, 2150 µL of 5 M NaCl, and filled to 4 mL with MilliQ water) and incubated for 15 min at 48 °C. Then the cells were air-dried at room temperature under dark for 2–4 h and then counterstained with 4,6-diamidino-2-phenylindole (DAPI) (final concentration of 10 mg/L) mounted in Citifluor (antifading agent). Image acquisition was done using confocal laser scanning microscopy (CLSM; Leica, TCS SPE). Duplicate analysis was conducted for each sample, and for each sample, two wells were prepared for imaging. At least 10 random locations were imaged from each well. The images were processed using the Image J and Leica software^58^.

### Reporting summary

Further information on research design is available in the Nature Research Reporting Summary linked to this article.

## Supplementary information

Reporting Summary

Supplementary Information

## Data Availability

All data generated or analyzed during this study are included in this article and its Supplementary Information file.
