# Supplementary material for: Competition of two highly specialized and efficient acetoclastic electroactive bacteria for acetate in biofilm anode of microbial electrolysis cell
Source: NPJ Biofilms Microbiomes. 2021 May 31;7:47. doi: 10.1038/s41522-021-00218-3 (PMC8166840; doi:10.1038/s41522-021-00218-3)
Supplement: Supplementary file 1 — Reporting Summary [file 41522_2021_218_MOESM1_ESM.pdf]

nature research

Corresponding author(s): Pascal Saikaly and Krishna Katuri

Last updated by author(s): Mar 20, 2021

Reporting Summary

Nature Research wishes to improve the reproducibility of the work that we publish. This form provides structure for consistency and transparency in reporting. For further information on Nature Research policies, see our Editorial Policies and the Editorial Policy Checklist.

Statistics

For all statistical analyses, confirm that the following items are present in the figure legend, table legend, main text, or Methods section.

n/a	Confirmed	
☐	☒	The exact sample size (*n*) for each experimental group/condition, given as a discrete number and unit of measurement
☐	☒	A statement on whether measurements were taken from distinct samples or whether the same sample was measured repeatedly
☒	☐	The statistical test(s) used AND whether they are one- or two-sided<br>*Only common tests should be described solely by name; describe more complex techniques in the Methods section.*
☒	☐	A description of all covariates tested
☒	☐	A description of any assumptions or corrections, such as tests of normality and adjustment for multiple comparisons
☒	☐	A full description of the statistical parameters including central tendency (e.g. means) or other basic estimates (e.g. regression coefficient) AND variation (e.g. standard deviation) or associated estimates of uncertainty (e.g. confidence intervals)
☐	☒	For null hypothesis testing, the test statistic (e.g. F, t, r) with confidence intervals, effect sizes, degrees of freedom and P value noted<br>*Give P values as exact values whenever suitable.*
☒	☐	For Bayesian analysis, information on the choice of priors and Markov chain Monte Carlo settings
☒	☐	For hierarchical and complex designs, identification of the appropriate level for tests and full reporting of outcomes
☒	☐	Estimates of effect sizes (e.g. Cohen's *d*, Pearson's *r*), indicating how they were calculated

Our web collection on statistics for biologists contains articles on many of the points above.

Software and code

Policy information about availability of computer code

Data collection	Data from MEC analysis was collected with EC-lab V 10.4 software of the VMP3 potentiostat (BioLogic Science Instruments, USA). Data from CLSM Leica SP7 was collected with LAS X Life Science software.
Data analysis	R.v. 3.3.4 using the R-studio environment version 1.0.153, EC-lab V 10.4, image J (Leica software).

For manuscripts utilizing custom algorithms or software that are central to the research but not yet described in published literature, software must be made available to editors and reviewers. We strongly encourage code deposition in a community repository (e.g. GitHub). See the Nature Research guidelines for submitting code & software for further information.

Data

Policy information about availability of data

All manuscripts must include a data availability statement. This statement should provide the following information, where applicable:

- Accession codes, unique identifiers, or web links for publicly available datasets
- A list of figures that have associated raw data
- A description of any restrictions on data availability

All data generated or analyzed during this study are included in this article and its Supplementary Information file.

Field-specific reporting

Please select the one below that is the best fit for your research. If you are not sure, read the appropriate sections before making your selection.

☒ Life sciences ☐ Behavioural & social sciences ☐ Ecological, evolutionary & environmental sciences

For a reference copy of the document with all sections, see nature.com/documents/nr-reporting-summary-flat.pdf

Life sciences study design

All studies must disclose on these points even when the disclosure is negative.

Sample size	No statistical methods were used to predetermine the sample size. In all experiments, two biological replicates were used, unless mentioned otherwise. Reproducible data possible due to robust nature of anodic biofilm under fixed imposed anode potential.
Data exclusions	No data were excluded from the analysis
Replication	All experiments were independently in duplicate reactors, and the data is reproducible.
Randomization	The experiments were not randomized, since all analyses concerned to specific enrichment cultures
Blinding	Investigations were not blinded to group allocation during the data collection or analysis

Reporting for specific materials, systems and methods

We require information from authors about some types of materials, experimental systems and methods used in many studies. Here, indicate whether each material, system or method listed is relevant to your study. If you are not sure if a list item applies to your research, read the appropriate section before selecting a response.

Materials & experimental systems

n/a	Involved in the study	
☒	☐	Antibodies
☒	☐	Eukaryotic cell lines
☒	☐	Palaeontology and archaeology
☒	☐	Animals and other organisms
☒	☐	Human research participants
☒	☐	Clinical data
☒	☐	Dual use research of concern

Methods

n/a	Involved in the study	
☒	☐	ChIP-seq
☐	☒	Flow cytometry
☒	☐	MRI-based neuroimaging

Flow Cytometry

Plots

Confirm that:

☐ The axis labels state the marker and fluorochrome used (e.g. CD4-FITC).

☐ The axis scales are clearly visible. Include numbers along axes only for bottom left plot of group (a 'group' is an analysis of identical markers).

☐ All plots are contour plots with outliers or pseudocolor plots.

☒ A numerical value for number of cells or percentage (with statistics) is provided.

Methodology

Sample preparation	Samples (200 µL) were transferred to a sterile Eppendorf tube and incubated at 35 °C for 10 min prior to staining with SYBR Green I (2 µL of 100× stock solution in 200 µL sample), vortexed, and then incubated again at 35 °C for 10 min. Samples (200 µL) were then transferred to a 96-well plate for cell counting. Concurrently, another 200 µl of the samples were stained with propidium iodide (2 µL of 100× stock solution) in combination with SYBR Green I (2 µL of 100× stock solution) to find live and dead (membrane-compromised) cells according to the same protocol used for total bacterial cell count. Flow cytometer equipped with a 50 mW laser having a fixed emission wavelength of 488 nm was used to measure the cell count. Fluorescence intensity was collected at FL1 = 533 ± 30 nm, FL3 > 670 nm, sideward and forward scattered light intensities were obtained as well. Electronic gating was used to select SYBR green I as well as propidium iodide and SYBR Green I staining labelled signals for quantifying total bacterial as well as live and dead cells following the procedure described earlier (Hammes and Egli, 2005). All data were processed with the BD Accuri CFlow® software. Unless specified otherwise, the

bacterial counts represent the live cell number.

Instrument	Accuri C6
Software	BD AccuriTM C6 software
Cell population abundance	Flow cytometry used to quantify the cell number but not sort the different species in a mixed population. Experiments conducted with pure cultures and were acquired from DSMZ, Germany. The purity of the cultures tested with 16s rRNA based analysis.
Gating strategy	Electronic gates used for separate bacterial cells from background carried out as suggested in Hammes et al., Water Research, 42, 269-277.

☐ Tick this box to confirm that a figure exemplifying the gating strategy is provided in the Supplementary Information.
