# Supplementary material for: Competition of two highly specialized and efficient acetoclastic electroactive bacteria for acetate in biofilm anode of microbial electrolysis cell
Source: NPJ Biofilms Microbiomes. 2021 May 31;7:47. doi: 10.1038/s41522-021-00218-3 (PMC8166840; doi:10.1038/s41522-021-00218-3)
Supplement: Supplementary file 2 — Supplementary Information [file 41522_2021_218_MOESM2_ESM.pdf]

Supplementary information

Competition of two highly specialized and efficient acetoclastic electroactive bacteria for acetate in biofilm anode of microbial electrolysis cell

Veerraghavulu Sapireddy†, Krishna P. Katuri†*, Ali Muhammad, and Pascal E. Saikaly*

Biological and Environmental Sciences and Engineering Division, Water Desalination and Reuse Center, King Abdullah University of Science and Technology, Thuwal 23955-6900, Saudi Arabia.

†Equal contribution

*Corresponding authors: krishna.katuri@kaust.edu.sa; pascal.saikaly@kaust.edu.sa

Supplementary Table 1. Normalized number of *D. acetexigens* and *G. sulfurreducens* cells extracted from the anode biofilm of a co-culture MEC reactor fed with synthetic medium containing acetate ($MEC_{co\text{-}culture\text{-}NaAc\text{-}N2}$).

**Time (h)**	**Cells/cm^2**		**Relative abundance (%)**	
	G. sulfurreducens	***D. acetexigens***	***G. sulfurreducens***	***D. acetexigens***
24	8.5E+04 ± 5.1E+03	2.8E+04 ± 3.1E+03	75 ± 3.2	25 ± 3.2
72	1.2E+06 ± 5.5E+03	1.1E+07 ± 4.1E+03	10.4 ± 0.04	89.5 ± 0.1
120	7.0E+05 ± 4.7E+03	2.0E+07 ± 1.6E+03	3.4 ± 0.02	96.5 ± 0.02
240	8.0E+05 ± 4.2E+04	3.2E+07 ± 2.3E+03	2.4 ± 0.1	97.5 ± 0.1
360	7.1E+05 ± 2.0E+04	3.4E+07 ± 9.1E+02	2 ± 0.05	98 ± 0.05
480	6.6E+05 ± 2.3E+03	3.3E+07 ± 1.6E+03	1.9 ± 0.01	98 ± 0.01

Supplementary Table 2. Normalized number of *D. acetexigens* and *G. sulfurreducens* cells extracted from the anode biofilm of a co-culture MEC reactor fed with domestic wastewater ($MEC_{co\text{-}culture\text{-}WW\text{-}N2}$).

**Time (h)**	***G. sulfurreducens* (cells/cm^2)**	***D. acetexigens* (cells/cm^2)**	**Total bacteria (cells/cm^2)**	***G. sulfurreducens* (%)**	***D. acetexigens* (%)**	***G. sulfurreducens + D. acetexigens* (%)**
120	8.2E+06 ± 2.9E+05	1.6E+07± 1.3E+06	3.8E+07 ± 7.6E+04	21.7 ± 0.7	44 ± 3.6	66 ± 2.8
360	1.1E+07± 1.4E+06	2.5E+07± 2.8E+06	4.6E+07 ± 3.5E+06	24 ± 1.1	55 ± 2	79 ± 3.1

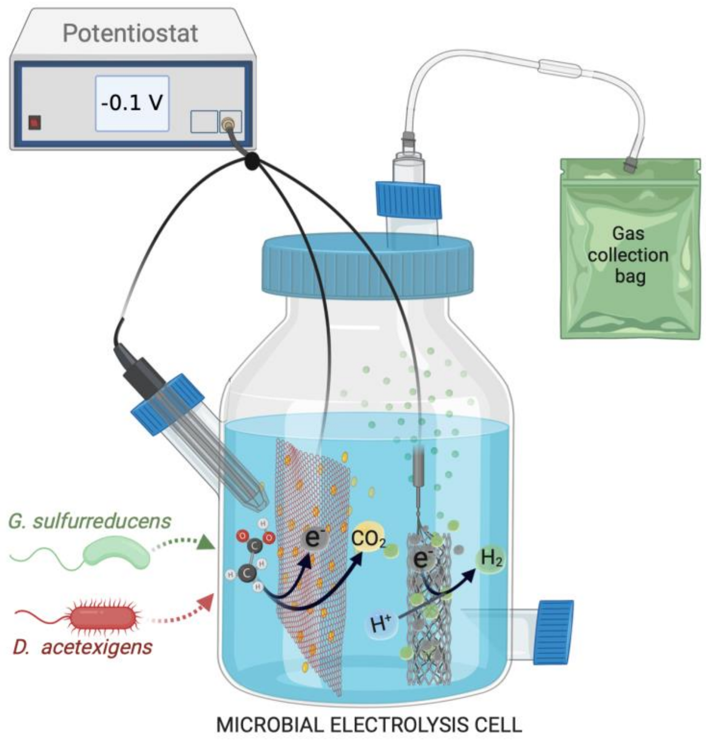

Supplementary Fig. 1. Schematic diagram of MEC used for conducting the experiments.

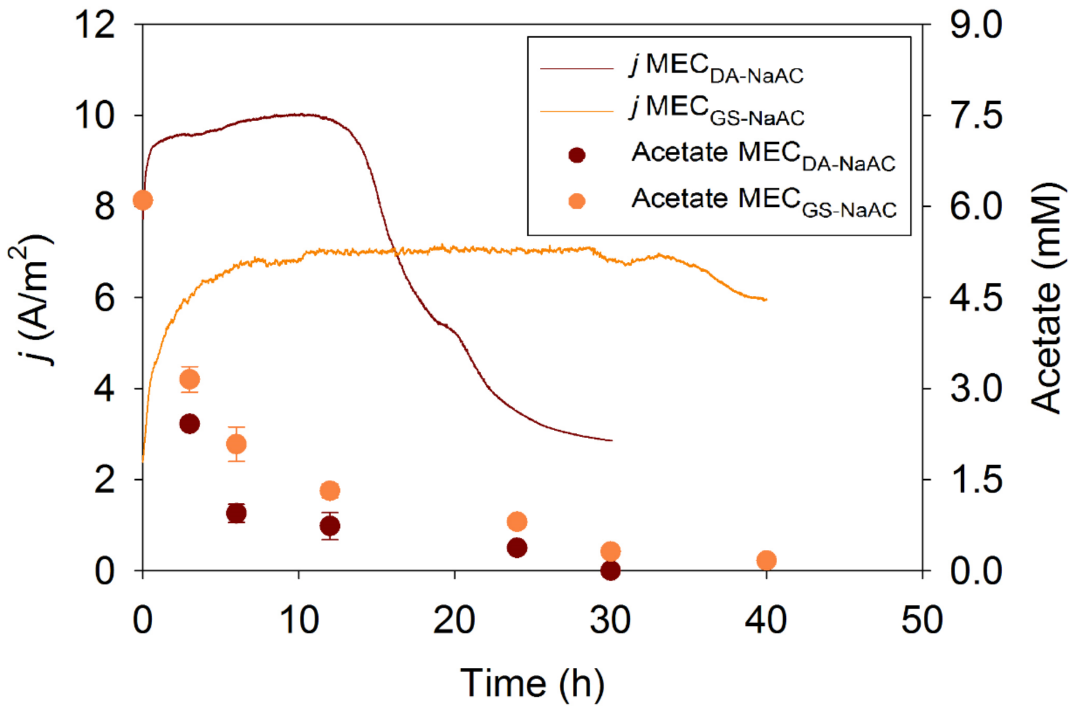

Supplementary Fig. 2. Current density and acetate consumption profile during batch 6 of operation for *D. acetexigens* ($MEC_{DA\text{-}NaAc}$) and *G. sulfurreducens* ($MEC_{GS\text{-}NaAc}$) biofilms under a set anode potential of –0.1 V vs Ag/AgCl. The current densities represent the average of triplicate MECs.

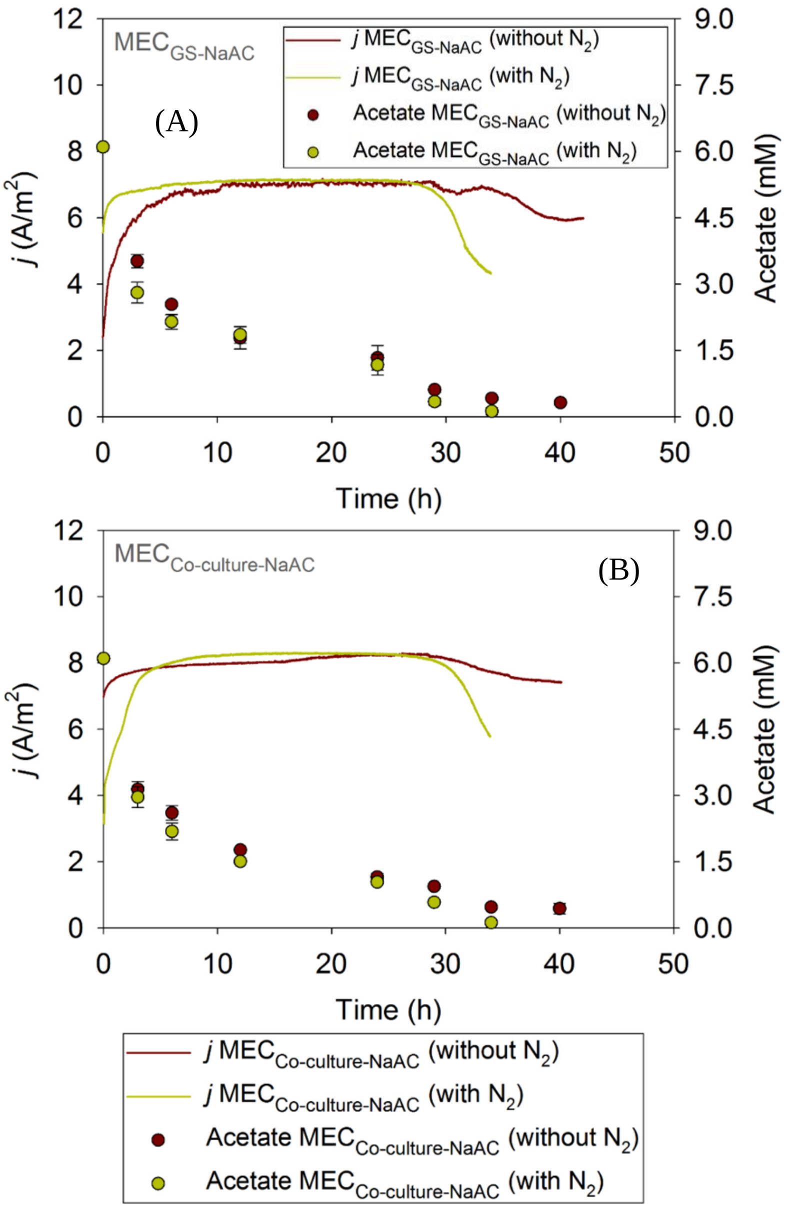

Supplementary Fig. 3. Amperometric response and acetate consumption during a batch operation of (A) monoculture MEC with *G. sulfurreducens* (MEC$_{GS-NaAc}$) and (B) co-culture MECs (MEC$_{co-culture-NaAc}$) with and without N_2 purging in the reactor headspace.

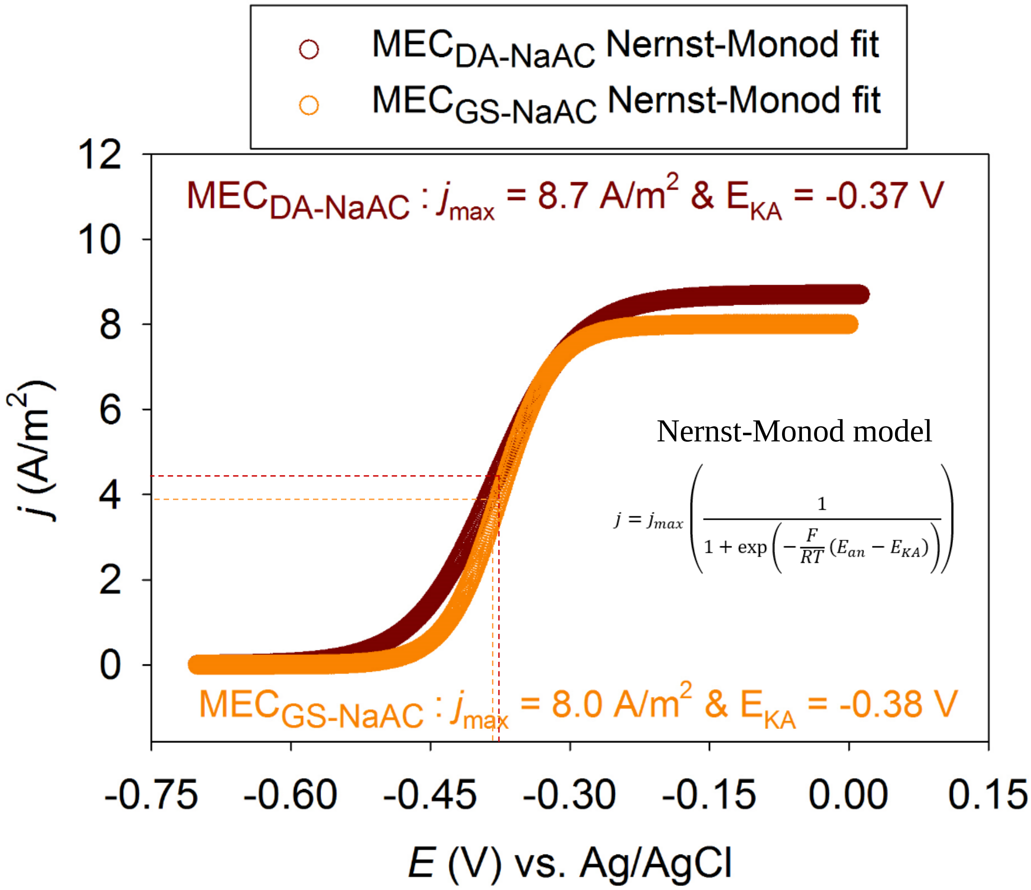

Supplementary Fig. 4. Comparison of Nernst-Monod kinetics of acetate (6 mM) oxidizing *D. acetexigens* and *G. sulfurreducens* biofilms (200 h aged) developed in MEC under a set anode potential (E_{an}) of –0.1 V vs Ag/AgCl. J_{max} and E_{KA} values used in the Nernst-Monod model were determined from Figure 2A. F is the Faraday constant (96,485 Coulombs/e^- mole), R is universal gas constant (8.314 J/mol K) and T is absolute temperature (K).

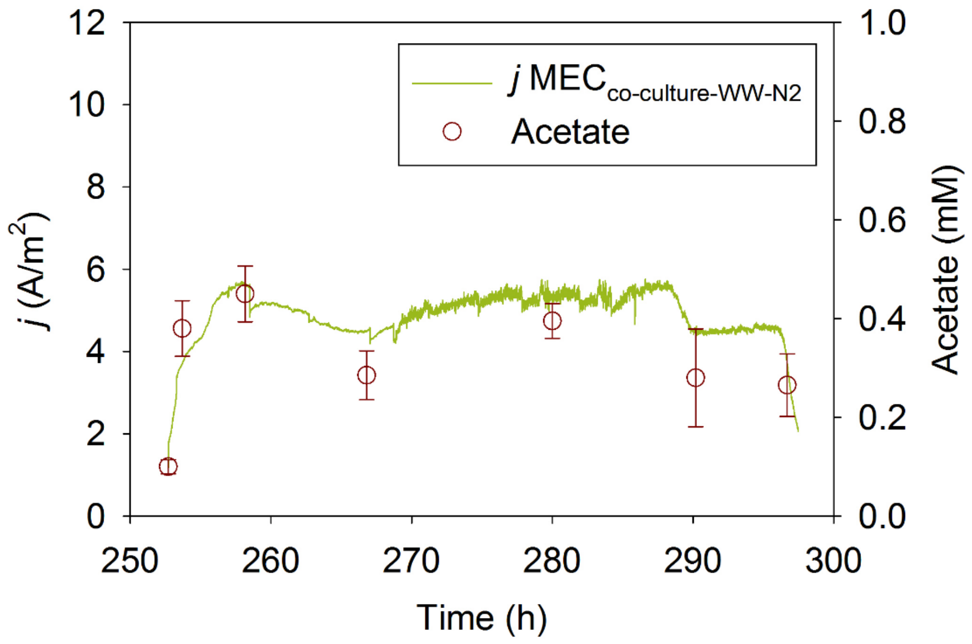

Supplementary Fig. 5. Acetate profile during a batch-cycle of operation in duplicate $MEC_{co\text{-}culture\text{-}WW\text{-}N2}$ reactors operated under –0.1 V vs Ag/AgCl set anode potential.

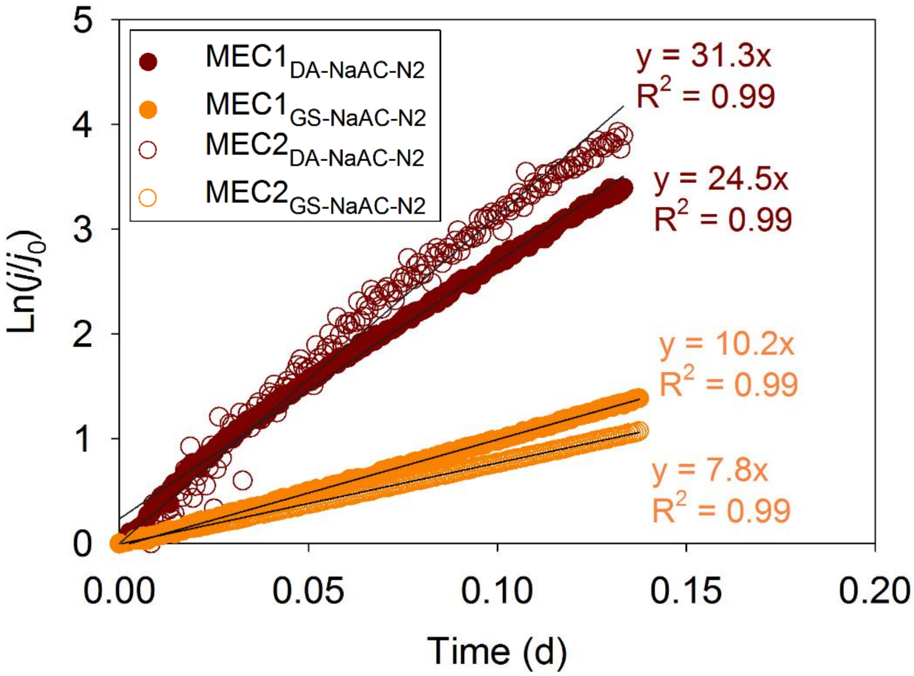

Supplementary Fig. 6. Estimation of maximum specific growth rate (μ_{max}) for duplicate growth experiments for each culture (i.e., *D. acetexigens* and *G. sulfurreducens*). The I/I_0 values of exponential part of Figure 7A were considered for the calculation of μ_{max} (d^{-1}), which is equal to the slope of the linear regression lines[2].

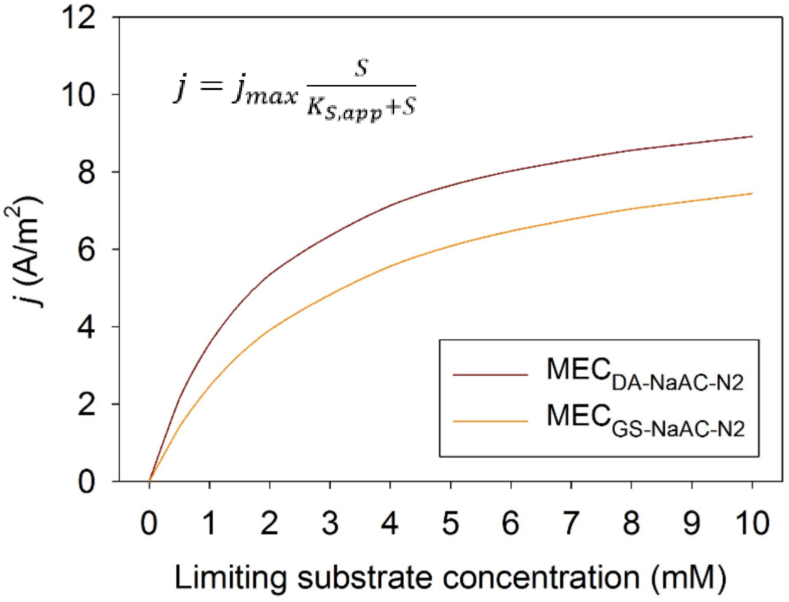

Supplementary Fig. 7. Simulated current density of *D. acetexigens* and *G. sulfurreducens* as a function of acetate concentration using the apparent kinetic parameters (j_{max} and $K_{S,\ app}$) calculated for each electroactive bacterium.

Supplementary References

1. Parameswaran, P., Bry, T., Popat, S.C., Lusk, B.G., Rittmann, B.E. and Torres, C.I. 2013. Kinetic, Electrochemical, and Microscopic Characterization of the Thermophilic, Anode-Respiring Bacterium *Thermincola ferriacetica*. Environmental Science & Technology 47(9), 4934-4940.
